# Forebrain-specific conditional calcineurin deficiency induces dentate gyrus immaturity and hyper-dopaminergic signaling in mice

**DOI:** 10.1186/s13041-022-00981-0

**Published:** 2022-11-22

**Authors:** Hideo Hagihara, Hirotaka Shoji, Mahomi Kuroiwa, Isabella A. Graef, Gerald R. Crabtree, Akinori Nishi, Tsuyoshi Miyakawa

**Affiliations:** 1 Division of Systems Medical Science, Center for Medical Science, Fujita Health University, Toyoake, Aichi 470-1192 Japan; 2 Department of Pharmacology, Kurume University School of Medicine, Kurume, Fukuoka 830-0011 Japan; 3 Department of Pathology, Stanford University of Medicine, Stanford, CA 94305 USA

**Keywords:** Calcineurin, Immature dentate gyrus, Dopamine receptor, cAMP, Intellectual disability, Mouse model

## Abstract

Calcineurin (Cn), a phosphatase important for synaptic plasticity and neuronal development, has been implicated in the etiology and pathophysiology of neuropsychiatric disorders, including schizophrenia, intellectual disability, autism spectrum disorders, epilepsy, and Alzheimer’s disease. Forebrain-specific conditional Cn knockout mice have been known to exhibit multiple behavioral phenotypes related to these disorders. In this study, we investigated whether Cn mutant mice show pseudo-immaturity of the dentate gyrus (iDG) in the hippocampus, which we have proposed as an endophenotype shared by these disorders. Expression of calbindin and GluA1, typical markers for mature DG granule cells (GCs), was decreased and that of doublecortin, calretinin, phospho-CREB, and dopamine D1 receptor (Drd1), markers for immature GC, was increased in Cn mutants. Phosphorylation of cAMP-dependent protein kinase (PKA) substrates (GluA1, ERK2, DARPP-32, PDE4) was increased and showed higher sensitivity to SKF81297, a Drd1-like agonist, in Cn mutants than in controls. While cAMP/PKA signaling is increased in the iDG of Cn mutants, chronic treatment with rolipram, a selective PDE4 inhibitor that increases intracellular cAMP, ameliorated the iDG phenotype significantly and nesting behavior deficits with nominal significance. Chronic rolipram administration also decreased the phosphorylation of CREB, but not the other four PKA substrates examined, in Cn mutants. These results suggest that Cn deficiency induces pseudo-immaturity of GCs and that cAMP signaling increases to compensate for this maturation abnormality. This study further supports the idea that iDG is an endophenotype shared by certain neuropsychiatric disorders.

**Supplementary Information:**

The online version contains supplementary material available at 10.1186/s13041-022-00981-0.

## Introduction

Endophenotype-oriented neuropsychiatric research has been increasingly considered important to improve the validity, reliability, and translatability of studies from animal models to human disease [1]. Accumulating evidence has indicated that pseudo-immaturity of the hippocampal dentate gyrus (DG) is found in a variety of animal models that exhibit behavioral abnormalities related to schizophrenia, intellectual disability (ID), autism spectrum disorders (ASD), epilepsy, and Alzheimer’s disease (AD) [2–13]. In the immature DG (iDG) phenotype, molecular expression patterns in the DG of adult mice resemble those of typically developing infants or adolescents with reduced mature marker (e.g., calbindin, glutamate receptor 1 [GluA1]) and increased immature marker (e.g., calretinin, doublecortin) expression. Importantly, pseudo-immaturity phenomena in the DG and other brain regions have been identified in human patients with schizophrenia, ASD, epilepsy, and AD, as assessed by decreased calbindin expression and/or increased calretinin expression and genome-wide gene expression patterns in post-mortem brain tissues [14–20], suggesting that pseudo-immaturity of particular brain regions is a phenomenon that can be observed across species. We have proposed iDG as a brain endophenotype shared by certain types of neuropsychiatric disorders [21].

Calcineurin (Cn) is a heterodimeric calcium/calmodulin-dependent serine/threonine protein phosphatase comprising Cnb regulatory and Cna catalytic subunits [22]. Cnb1, an isoform of Cnb, is the only regulatory subunit expressed in the brain, while several different Cna isoforms are expressed in the brain [22], with a particular abundance in the hippocampus, cortex, and striatum [23, 24]. Cn is known to be involved in a wide range of neuronal functions and development, such as *N*-methyl-D-aspartate (NMDA) receptor-dependent long-term depression in hippocampal CA1 neurons [25, 26], dendritic spine size dynamics in cortical neurons [26, 27], and axonal outgrowth in some types of embryonic neurons [29, 30]. Genetic studies have suggested an association between the genes encoding CNA isoforms and schizophrenia [31–34]. Recently, de novo mutations in *PPP3CA*, a gene encoding a CNA isoform, have been repeatedly reported as a cause of ID /developmental delay and epilepsy, which are often accompanied by autistic features [35–40]. More recent large-scale association studies have identified a number of genes that have common and rare variants associated with schizophrenia [41–43] and showed a nominal association for rare variants in PPP3CA with no genome-wide significance [41]. RNA sequencing analysis has revealed that expression of *CNB1* (*PPP3R1*) is reduced in the cortex of patients with ASD [44]. Mutant mice with forebrain neuron-specific deletion of *Cnb1* (Cn mutants) exhibited multiple behavioral abnormalities related to schizophrenia and other neuropsychiatric disorders, including hyper-locomotor activity (mania-like behavior), reduced nest-building activity (a mimic of negative-like symptoms [45]), and working memory deficits [24, 46], supporting the idea that alterations in CN signaling or in mechanism(s) supported by CN functions may be an important contributing factor in the pathogenesis of these diseases [46]. However, it remains unknown whether Cn deficiency causes neuronal pseudo-immaturity in the DG, and if so, what behaviors are associated with such an immature phenotype. In addition, it is unknown whether Cn mutants exhibit increased expression of Drd1, a shared feature among mouse models with the iDG phenotype [21], such as Camk2a^±^ mice and the mice chronically treated with the antidepressant fluoxetine [47, 48]. An increase in DRD1 expression has been observed in some cortical regions of patients with schizophrenia [49–51]. The current study addresses these questions using molecular expression pattern analyses, phosphorylation assays of Drd1 signaling substrates, and behavioral tests in combination with pharmacological manipulation to rescue the observed multi-level phenotypes in Cn mutant mice.

## Methods

A detailed description of the Materials and Methods is provided in the Additional file 1: Supplementary Materials and Methods.

### Animals

All animal experiments were approved by the Institutional Animal Care and Use Committee of Fujita Health University and Kurume University based on the Law for the Humane Treatment and Management of Animals and the Standards Relating to the Care and Management of Laboratory Animals and Relief of Pain. Every effort was made to minimize the number of animals used. The forebrain-specific Cnb deficient mice were generated by mating a male mouse homozygous for floxed Cnb (Cnb^flox/flox^) [52] with a female Cnb^flox/wild^ mouse carrying the calcium/calmodulin-dependent protein kinase II alpha-Cre transgene (Camk2a-Cre^±^) [53]. CNB floxed mice and Camk2a-Cre mice were backcrossed to C57BL6/J mice for at least seven and ten generations, respectively, before experimental intercrosses. The resulting genotypes were Camk2a-Cre^−/−^, Cnb^flox/wild^; Camk2a-Cre^±^, Cnb^flox/wild^; Camk2a-Cre^−/−^, Cnb^flox/flox^; and Camk2a-Cre^±^, Cnb^flox/flox^. We confirmed that the first three genotypes showed no significant difference in expression of mature/immature granule cell markers (Additional file 1: Fig. S1). Therefore, Camk2a-Cre^−/−^, Cnb^flox/wild^ mice, Camk2a-Cre^±^, Cnb^flox/wild^ mice, or Camk2a-Cre^−/−^, Cnb^flox/flox^ mice were used for the littermate control group and Camk2a-Cre^±^, Cnb^flox/flox^ mice were used for the Cnb deficient group. We used male and female adult mice (> 8 weeks old) in this study.

### Immunohistochemistry

Immunohistochemical analysis was performed as previously described [54, 55]. Primary antibodies used in this study are listed in Additional file 2: Table S1. Immunoreactivity to the antigen was visualized using Alexa Fluor 488-conjugated secondary antibody (Molecular Probes, Eugene, OR). Nuclear staining was performed with Hoechst 33,258 (Polysciences, Warrington, PA). We used a microscope (LSM 510 META; Zeiss, Göttingen, Germany) to obtain images of the stained sections. Three to seven sections from each animal were processed for semi-quantification analyses, and the averaged values were considered as one sample. Immunofluorescence intensity and the number of stained cells were counted in the indicated hippocampal regions, manually delineated using ZEN software (Zeiss) or ImageJ software (http://rsb.info.nih.gov/ij/) according to the mouse brain atlas [56]. Semi-quantification analyses were performed in the dorsal hippocampus (approximately from − 2.1 to − 1.6 mm from the bregma).

### Real-time quantitative PCR

We used 11-week-old mice for the analysis [57]. The following primers were used: dopamine d1 receptor (Drd1a) (1–124), 5′-ATGGCTCCTAACACTTCTACCA and 5′-GGGTATTCCCTAAGAGAGTGGAC; tryptophan 2,3-dioxygenase (Tdo2) (1–105), 5′-ATGAGTGGGTGCCCGTTTG and 5′-GGCTCTGTTTACACCAGTTTGAG; desmoplakin (Dsp) (7–113), 5′-GCTGAAGAACACTCTAGCCCA and 5′-ACTGCTGTTTCCTCTGAGACA; and β-actin (851–962), 5′-AGTGTGACGTTGACATCCGTA and 5′-GCCAGAGCAGTAATCTCCTTCT. Ct values used were the means of two or three replicates (Additional file 2: Table S2).

### DNA microarray and data processing

Dissection of the mouse DG [57] and microarray experiments were performed as described previously [54]. Thirteen to 14 weeks old Cn mutant and control mice were used. The raw microarray data were deposited in the GEO database under accession number GSE175896, and the processed data is available in Additional file 2: Table S3. The following microarray datasets were also used: the DG of postnatally developing mice (GSE113727) [17, 58], the DG of Camk2a^±^ mice [57], the DG of Hivep2 (also called Schnurri-2) knockout (KO) mice (GSE42777) [3], and the DG of mice chronically treated with an antipsychotics fluoxetine (GSE118669) [58].

Using the expression values, we calculated fold changes and t-test P-values between experimental mice and the corresponding control mice. The average value of 8, 11, 14, 17, 21, and 25 days old mice was divided by that of 29 days old mice, respectively. Genes (or transcripts) with absolute fold change > 1.2 and P‐value < 0.05 (without correction for multiple testing) were imported to the web‐based bioinformatics tool BaseSpace (Illumina, San Diego, CA; https://basespace.illumina.com) [59] according to the manufacturer's instructions. Subsequently, the gene expression patterns of the two given gene sets were statistically compared using BaseSpace [17, 60, 61]. Using the bioinformatics tool, similarities were examined using the Running Fisher algorithm, a nonparametric rank‐based statistical method, in which information regarding the rank based on the absolute value of fold change and the direction of gene expression changes within each gene set was considered [59]. The greater the similarity in gene expression patterns between the two conditions, the lower the resulting overlap P‐value. Details of the algorithm have been described previously [3].

### Pathway enrichment analysis

Pathways/biogroups enriched in the differentially expressed genes (DEGs) were determined through a combination of rank‐based enrichment statistics and biomedical ontologies using BaseSpace [59]. Pathways/biogroups from GO and canonical pathways of Broad MSigDB were included in this analysis.

### Drug treatment

Eight-week-old mice were administered with 2 mg/kg of rolipram (R6520; Sigma-Aldrich, St Louis, MO) or vehicle (1% dimethyl sulfoxide in saline) intraperitoneally once daily at a volume of 5 ml/kg for 3 weeks. On the next day of the final treatment, mice were processed for immunohistochemical analyses, slice experiments, and behavioral tests. Mice for behavioral testing were kept treated with rolipram, which was administered at the end of the experiments every day, during behavioral testing.

### Slice experiments

Preparation of DG slices and immunoblotting were performed as described previously [48]. Briefly, the regions of DG were dissected from 350-μm-thick coronal slices between − 1.4 and − 3.8 mm from the bregma. The DG slices were incubated with or without SKF81297 (Sigma-Aldrich) for 10 min and processed for immunoblotting analysis. Eight mice were analyzed for each condition. Primary antibodies used in this study are listed in Additional file 2: Table S1.

### Behavioral tests

The open field test, T-maze test, startle response/prepulse inhibition (PPI) test, and nest building test was performed as described previously [3, 46, 62–64].

### Statistical analysis

The data were analyzed by Student’s t-test, one-way ANOVA, or two-way ANOVA followed by Sidak’s multiple comparison test using R or Prism 8 version 8.4.2 (GraphPad Software, Inc., San Diego, CA). We used the nonparametric tests (Mann Whitney test and Friedman test) when the homogeneity of variance and normality assumptions were not met. In the behavioral test battery, we defined “study-wide significance” as the statistical significance that survived false discovery rate (FDR) correction for 14 indices in the comparison between Mut + Rol and Mut + Veh groups and “nominal significance” as the one that achieved a statistical significance in an index (*P* < 0.05) but did not survive this correction.

## Results

### Cn deficiency induces immature dentate gyrus phenotype as assessed by typical neuronal maturation markers

**Fig. 1:**
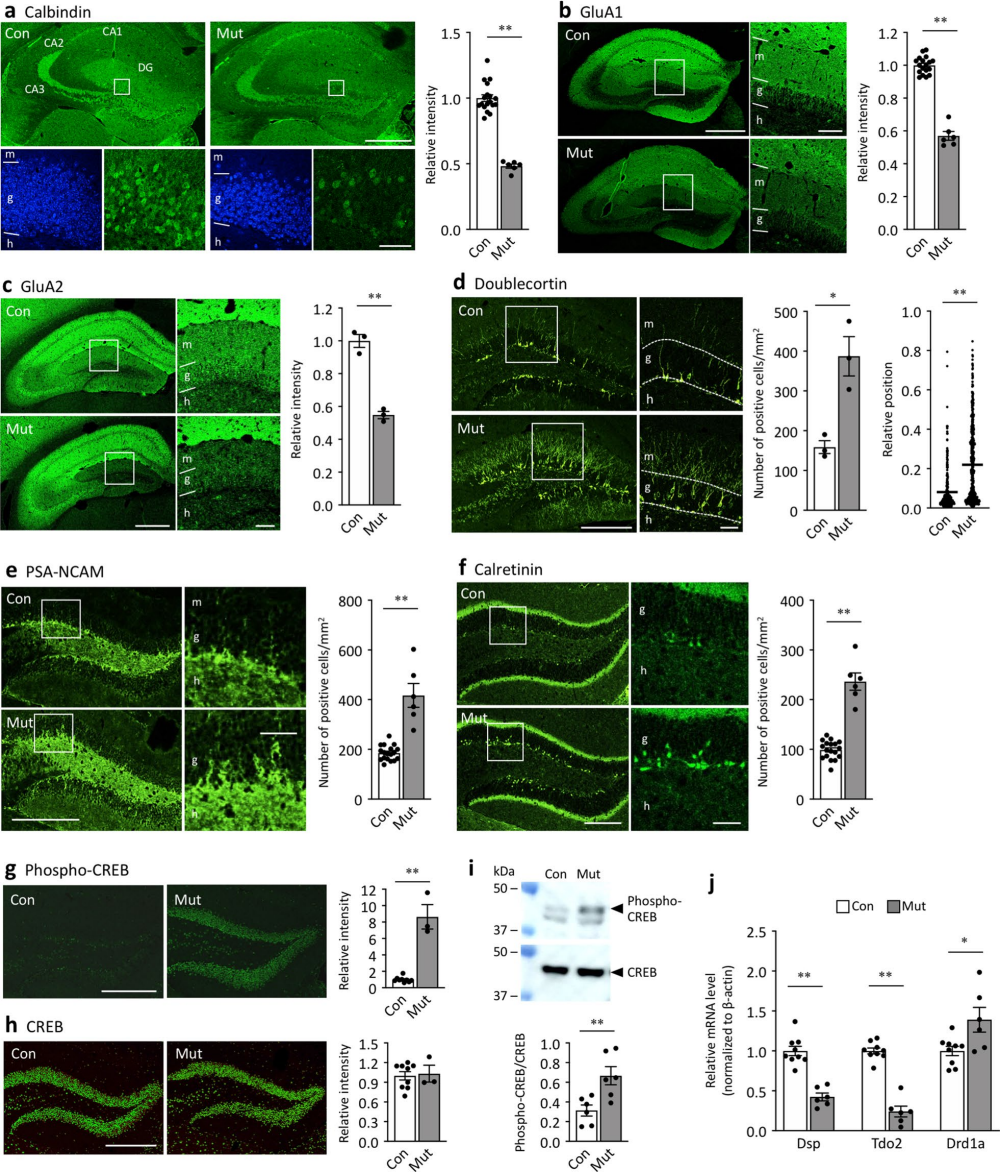
Immature dentate gyrus phenotype assessed using typical molecular markers in Cn mutant mice. **a**–**h** Immunostaining images and quantified bar graphs of calbindin (**a**; t = 11.20, df = 22, *P* < 0.0001), GluA1 (**b**; t = 16.66, df = 22, *P* < 0.0001), GluA2 (**c**; t = 10.00, df = 4, *P* = 0.0006), doublecortin (**d**; left graph: t = 4.39, df = 4, *P* = 0.012; right graph: t = 11.66, df = 826, *P* < 0.0001), PSA-NCAM (**e**; F test: *P* < 0.0001; Mann Whitney test: *P* < 0.0001), calretinin (**f**; t = 11.23, df = 22, *P* < 0.0001), phospho-CREB (**g**; F test: *P* < 0.0001; Mann Whitney test: *P* = 0.0091), and CREB (**h**; t = 0.24, df = 10, *P* = 0.81). **i** Western blot images and quantified bar graphs of phospho-CREB and CREB. Graphs show relative levels of phospho-CREB over total CREB. *P* = 0.0085. The original blots are included in Additional file 1: Fig. S3. **j** Expression levels of *Dsp*, *tdo2*, and *Drd1a* mRNA (*Dsp*: t = 6.95, df = 13, *P* < 0.0001; *Tdo2*: t = 10.64, df = 14, *P* < 0.0001; *Drd1a*: t = 2.71, df = 13, *P* = 0.018). Bar graphs show means ± SEM. Each dot represents one mouse, except for the right graph in **d**. The positions of doublecortin-positive cells are shown as relative values between the subgranular layer (0) and the border with the molecular layer (1) (**d**, right graph; each dot represents one cell). Scale bars: **a** upper panels 500 μm, lower panels 50 μm; **b**, **c** left panels 500 μm, right panels 100 μm; **d**, **e** left panels 300 μm, right panels 50 μm; **f** left panels 200 μm, right panels 50 μm; **g**, **h** 50 μm. Relative intensity means the ratio of the immunoreactive intensity obtained from the mutants to that of the controls. *P < 0.05, **P < 0.01, Students t-test. Con, control mice; Mut, Cn mutant mice

We first examined the expression patterns of typical markers of mature and immature GCs in the DG. The immunoreactive intensity of calbindin, a mature GC marker, was drastically decreased in the GC layer of Cn mutants (Fig. 1a). The number of calbindin-positive cells was also decreased in the GC layer of Cn mutants (Con, 4078.8 ± 172.6 cells/mm^2^; Mut, 1285.2 ± 341.0 cells/mm^2^; *P* < 0.0001). Immunoreactive intensity of GluA1 and GluA2, other markers of mature GC [65], was decreased in the GC layer (Fig. 1b and c) and molecular layer (Additional file 1: Fig. S2) of Cn mutants, indicating the reduced expression of GluA1 and GluA2 in both soma and dendrites of GC. The number of Hoechst-stained nuclei in the GC layer was not significantly different between genotypes (mutants: 112.64 ± 3.59 cells/mm^2^, controls: 113.43 ± 5.10 cells/mm^2^; *P* = 0.90), indicating that the decreased expression of those mature GC markers in Cn mutants was not due to the loss of GCs. Expression levels of mRNA for other mature GC markers, *Dsp* and *Tdo2* [66], were also significantly lower in the DG of Cn mutants (Fig. 1i). In contrast, the expression of doublecortin (Fig. 1d), PSA-NCAM (Fig. 1e), and calretinin (Fig. 1f), markers for progenitors or immature GCs, was higher in the Cn mutants. Doublecortin-positive cells were observed within the GC layer in Cn mutants, whereas in controls, such cells were mostly located in the subgranular zone, where GCs proliferate and differentiate into new neurons (Fig. 3d). We also found that the expression of phospho-cAMP-dependent response element binding protein (CREB), which is predominantly expressed in immature GCs and located in the inner GC layer in the normal adult DG [67–69], was increased throughout the GC layer in Cn mutants (Fig. 1g), while the expression levels of total CREB were almost normal (Fig. 1h). Western blot analysis confirmed increased CREB phosphorylation in the DG of Cn mutants (Fig. 1i, Additional file 1: Fig. S3). We also found that the expression of *Drd1a* mRNA was increased in the DG of Cn mutants compared to that in controls (Fig. 1j).

**Fig. 2:**
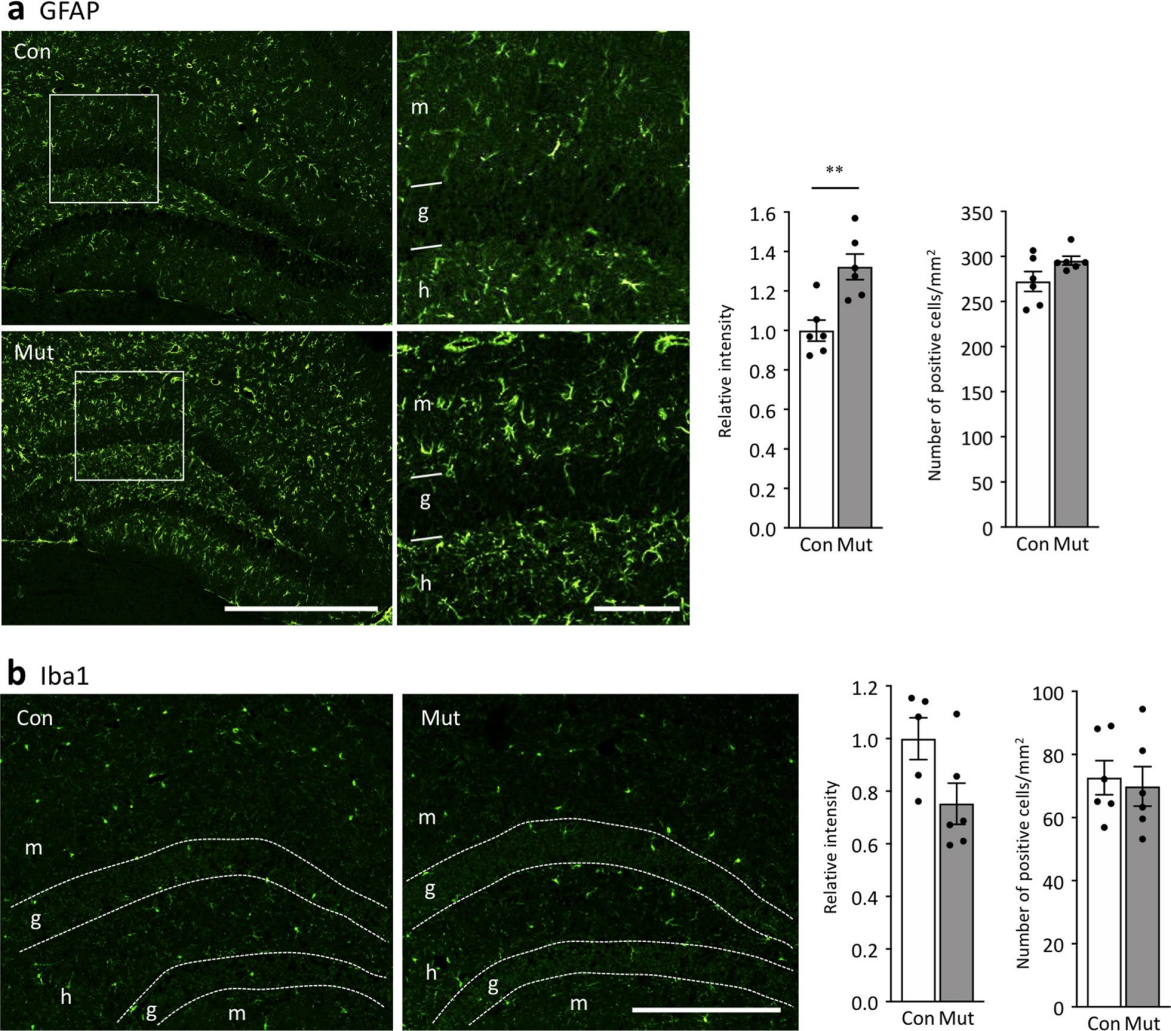
Activation of astrocytes in the DG of Cn mutant mice. Immunostaining images and quantified bar graphs of GFAP (**a**; left graph: t = 3.84, df = 10, *P* = 0.0032; right graph: t = 1.93, df = 10, *P* = 0.083) and Iba1 (**b**; left graph t = 2.20, df = 9, *P* = 0.055; right graph t = 0.33, df = 10, *P* = 0.75). Bar graphs show means ± SEM. Each dot represents one mouse. Scale bars: **a** left panels 500 μm, right panels 100 μm; **b** 300 μm. Relative intensity means the ratio of the immunoreactive intensity obtained from the mutants to that of the controls. **P < 0.01, Students t-test

An additional feature common to iDG mouse models is the activation of astrocytes in the DG [21]. Studies in human post-mortem brains have suggested activation of astrocytes in the brain of patients with neuropsychiatric disorders, including ASD [70, 71], ID [72], epilepsy [73, 74], schizophrenia [75, 76], and AD [77], which are considered inflammatory conditions of the brain [75]. In Cn mutants, the expression of glial fibrillary acidic protein (GFAP), an astrocytic marker, increased significantly in the molecular layer of the DG, while the number of GFAP-positive cells was not different from that in controls (Fig. 2a). Moreover, the expression of S100 calcineurin binding protein B (S100B), another marker of astrocytes, was increased significantly in the molecular layer of the DG, while the number of S100B-positive cells was not different from that in controls (Additional file 1: Fig. S4). We found that neither expression of the microglial marker Iba1 nor the number of Iba1-positive cells was changed in Cn mutants (Fig. 2b). These results suggest astrogliosis with no apparent microgliosis in the DG of Cn mutants. Thus, Cn mutants showed the iDG phenotype as assessed by typical marker expressions that were common to previously identified mouse models with iDG [21].

### Transcriptomic evidence for the immaturity of the DG in Cn mutant mice

**Fig. 3:**
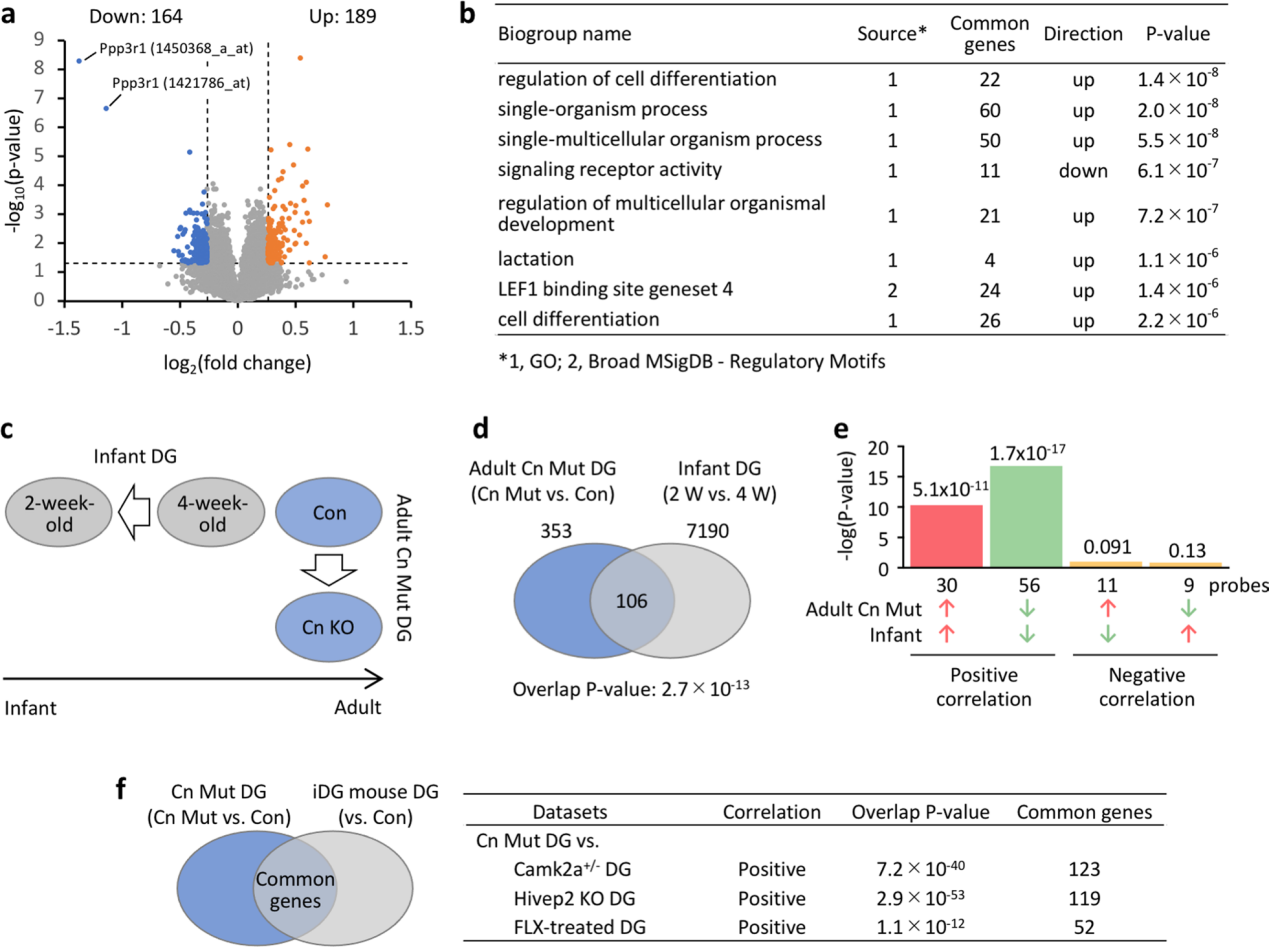
Transcriptomic evidence for the immature dentate gyrus phenotype in Cn mutant mice. **a** Volcano plot showing the differentially expressed genes (DEGs) identified by microarray analysis. **b** Pathway enrichment analysis of the DEGs using BaseSpace. Full list is available in the Additional file 2: Table S4. **c** The gene expression pattern in the DG of adult Cn mutant mice (mutants compared to controls) was compared with typically developing infant mice (2-week-old vs. 4-week-old wild-type mice). **d** Venn diagram illustrating the overlap in transcriptome‐wide gene expression changes in the DG of adult Cn mutant mice and infant mice. **e** P‐values of overlap between the adult Cn mutant mice and infant mice in the DG datasets. Bar graphs illustrate the P‐values of overlap of genes upregulated (red arrows) or downregulated (blue arrows) by each condition, between the two conditions. **f** Overlap P-values and the number of common genes/transcripts responsive to both conditions for each pair of interest. iDG mouse, mouse with immature dentate gyrus (iDG) phenotypes

Next, we evaluated the iDG phenotype in Cn mutants at a genome-wide gene expression level. Microarray analysis revealed that of 45,037 transcripts tested, 353 were differentially expressed in the DG of Cn mutants compared to controls (absolute fold change > 1.2, *P* < 0.05, without correction for multiple tests; Fig. 3a). *Cnb1* (*Ppp3r1*) displayed the lowest value among the differentially expressed genes (DEGs) (Additional file 2: Table S3). Pathway analysis showed that terms related to cell proliferation and development were enriched in the DEGs (Fig. 3b; Additional file 2: Table S4), supporting the idea of maturation abnormalities in the DG of Cn mutants.

To further define the iDG phenotype in Cn mutants, we used publicly available microarray datasets to conduct a comparative transcriptomic analysis. This analysis examined whether, or to what extent, overall gene expression patterns were similar between adult Cn mutants and typically developing infant mice (Fig. 3c). Transcriptome datasets were compared using the Running Fisher test, a non-parametric rank-based statistical method, which calculates overlap P-values between two given gene sets in consideration of fold-change-based rank and the direction of changes [59]. In this analysis, we identified a highly significant overlap P-value with a positive correlation between the DG of adult Cn mutants (mutants vs. controls) and the DG of infant mice (2-week-old vs. 1-month-old) (*P* = 2.7 × 10^–13^; Fig. 3d and e, Additional file 2: Table S5), indicating a significant similarity in the gene expression patterns between them. Furthermore, the patterns of gene expression changes in Cn mutants were similar to those in Hivep2 KO mice [3], Camk2a^±^ mice [2], and fluoxetine-treated mice [47, 48], and other mouse models with iDG (Fig. 3f). These results confirmed the iDG phenotype in Cn mutants from a transcriptomic standpoint.

### Chronic rolipram treatment ameliorates iDG phenotype and nest-building behavior

Increased expression of Drd1a, which stimulates intracellular cAMP signaling, is a common feature found in previously identified mouse models with iDG [20], and an increase was also found in Cn mutants (Fig. 1i). We also found an increase in CREB phosphorylation (Fig. 1g and h), which are known to be increased by cAMP/protein kinase A (PKA) [78], while CREB is suggested to be not a direct target of Cn [79]. These results suggest that cAMP signaling is elevated in the DG of Cn mutants. We previously found that chronic treatment with rolipram, a cAMP-specific phosphodiesterase inhibitor that elevates intracellular cAMP levels (in combination with ibuprofen), rescued the iDG phenotype in Hivep2 KO mice [3], raising the possibility that the increased cAMP signaling in Cn mutants is due to a compensatory mechanism. To determine whether the increased cAMP signaling is related to compensatory or pathological mechanisms underlying the iDG and behavioral phenotypes, we investigated the effect of rolipram on the phenotypes of Cn mutants.

**Fig. 4:**
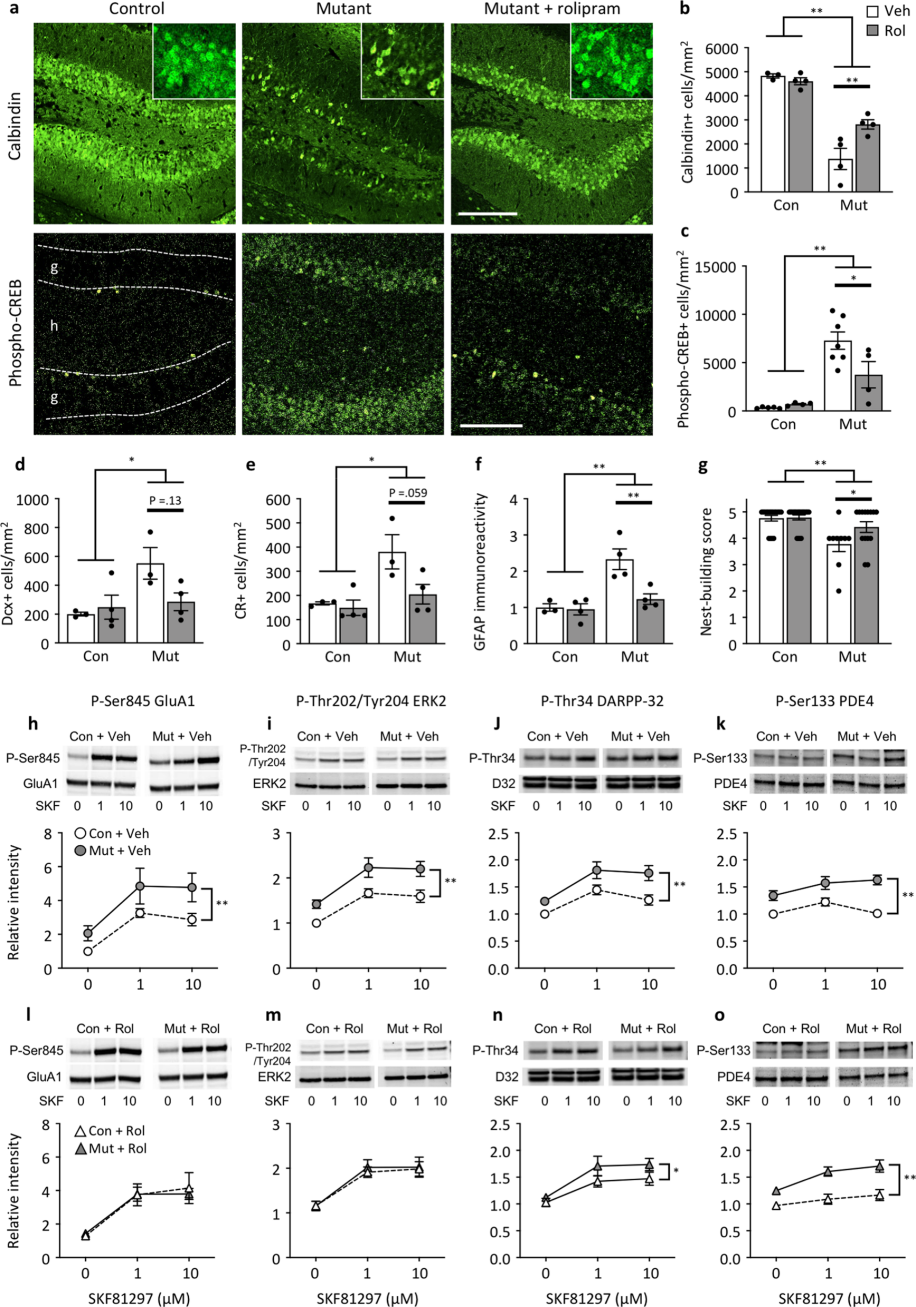
Rescue of immature dentate gyrus phenotype and impaired nest building behavior by chronic rolipram treatment in Cn mutant mice. **a** Immunostaining images of calbindin (upper panels) and phospho-CREB (lower panels). Scale bars, 200 μm. G, granule cell layer; h, hilus. **b**–**g** Bar graphs showing the number of calbindin-positive cells (**b**; Bartlett test: genotype, *P* = 0.0037, drug, *P* = 0.11; Friedman test: genotype effect: F(1, 11) = 33.52, *P* < 0.0001; drug effect: F(1, 11) = 13.84, *P* = 0.0034; interaction: F(1, 11) = 9.79, *P* = 0.0096), the number of phospho-CREB-positive cells (**c**; Bartlett test: genotype, *P* = 0.016, drug, *P* = 0.024; Friedman test: genotype effect: F(1, 16) = 45.15, *P* < 0.0001; drug effect: F(1, 16) = 7.37, *P* = 0.015; interaction: F(1, 16) = 3.59, *P* = 0.076), the number of doublecortin (Dcx)-positive cells (**d**; genotype effect: F(1,10) = 6.50, *P* = 0.029; drug effect: F(1, 10) = 2.04, *P* = 0.18; interaction: F(1, 10) = 4.22, *P* = 0.067), number of calretinin (CR)-positive cells (**e**; Bartlett test: genotype, *P* = 0.023, drug, *P* = 0.13; Friedman test: genotype effect: F(1, 10) = 10.81, *P* = 0.0082; drug effect: F(1, 10) = 6.15, *P* = 0.033; interaction: F(1, 10) = 5.22, *P* = 0.046), GFAP immunoreactivity (**f**; Bartlett test: genotype, *P* = 0.016, drug, *P* = 0.024; Friedman test: genotype effect: F(1, 11) = 33.52, *P* = 0.00012; drug effect: F(1, 11) = 12.14, *P* = 0.0051; interaction: F(1, 11) = 10.23, *P* = 0.0085), and nest-building score (g; genotype effect: F(1,55) = 17.70, *P* < 0.0001; drug effect: F(1, 55) = 4.45, *P* = 0.040; interaction: F(1, 55) = 3.82, *P* = 0.056). *P < 0.05, **P < 0.01, two-way ANOVA or Friedman test followed by multiple comparison test. Bar graphs show means ± SEM. Each dot represents one mouse. **h**–**o** Expression levels of P-Ser845 GluA1 (**h**, **l**), P-Thr202/Tyr204 ERK2 (**i**, **m**), P-Thr34 DARPP-32 (**j**, **n**), and P-Ser133 PDE4B1 (**k**, **o**) in the DG slices from vehicle (Veh)-treated mice (**h**–**k**) or rolipram (Rol)-treated mice (**l**–**o**) with or without preincubation with SKF81297. The data were normalized to total protein and values obtained with untreated slices from vehicle-treated control mice. The expression levels of total proteins were not altered significantly between genotypes or in response to SKF81297. The original blots are included in Additional file 1: Fig. S7. *P < 0.05, **P < 0.01, two-way ANOVA or Friedman test. **h** Bartlett test: genotype, *P* = 0.00075, SKF81297, *P* = 0.013; Friedman test: Genotype effect: F(1, 42) = 15.55, *P* = 0.00030; effect of SKF81297: F(2, 42) = 26.14, *P* < 0.0001; interaction: F(2, 42) = 0.45, *P* = 0.64. **i** Genotype effect: F(1, 42) = 23.11, *P* < 0.0001; effect of SKF81297: F(2, 42) = 18.57, *P* < 0.0001; interaction: F(2, 42) = 0.26, *P* = 0.77. **j** Genotype effect: F(1, 42) = 18.35, *P* = 0.0001; effect of SKF81297: F(2, 42) = 12.94, *P* < 0.0001; interaction: F(2, 42) = 0.78, *P* = 0.46. **k** Genotype effect: F(1, 36) = 42.55, *P* < 0.0001; effect of SKF81297: F(2, 36) = 4.07, *P* = 0.026; interaction F(2, 36) = 1.87, *P* = 0.17. **l** Genotype effect: F(1, 42) = 0.015, *P* = 0.90; effect of SKF81297: F(2, 42) = 15.41, *P* < 0.0001; interaction: F(2, 42) = 0.13, *P* = 0.88. **m** Genotype effect: F(1, 42) = 0.14, *P* = 0.71; effect of SKF81297: F(2, 42) = 19.15, *P* < 0.0001; interaction: F(2, 42) = 0.068, *P* = 0.93. **n** Genotype effect: F(1, 42) = 5.09, *P* = 0.0293; effect of SKF81297 F(2, 42) = 12.52, *P* < 0.0001; interaction: F(2, 42) = 0.39, *P* = 0.68. **o** Genotype effect: F(1, 36) = 36.62, *P* < 0.0001; effect of SKF81297: F(2, 36) = 7.04, *P* = 0.0026; interaction: F(2, 36) = 1.34, *P* = 0.27. Data are shown as means ± SEM

The rolipram treatment significantly increased the expression levels of the mature GC marker calbindin (*P* = 0.0057; Fig. 4a and b) and decreased the expression levels of the immature GC marker phospho-CREB (*P* = 0.033; Fig. 4a and c) in Cn mutants. Expression levels of GluA1 were not affected by rolipram treatment (Additional file 1: Fig. S5), and the number of doublecortin-positive (Fig. 4d) and calretinin-positive cells (Fig. 4e) tended to be lower following rolipram treatment in Cn mutants. These results suggest that chronic treatment with rolipram partially rescued the iDG phenotype in Cn mutants. Moreover, chronic rolipram treatment decreased the expression of the astrocytic marker, GFAP, in mutants (*P* = 0.010; Fig. 4f), which may be due to the anti-inflammatory effects of the drug [80].

A series of behavioral tests revealed that chronic rolipram treatment improved the impaired nest-building activity in Cn mutants at a nominal significance level (raw *P* = 0.025; Fig. 4g). Chronic treatment with rolipram did not significantly affect locomotor activity or anxiety-like behavior in the open field test (Additional file 1: Figs. S6a–S6d), prepulse inhibition (Additional file 1: Figs S6e and S6f), or working memory assessed using the T-maze spontaneous alternation task (Additional file 1: Fig. S6g). Thus, rolipram treatment selectively rescued impaired nesting behavior, which is considered to be associated with negative symptoms of psychiatric disorders [45].

### Increased Drd1a/PKA signaling activity in the DG of Cn mutant mice

Considering the increased expression of Drd1a in Cn mutants and the cAMP-modulating effect of rolipram, we investigated cAMP-dependent PKA signaling activity downstream of Drd1a to gain mechanistic insights into the rescue effects of rolipram. After the 3-week treatment of mice with rolipram or vehicle, we prepared DG slices and examined the phosphorylation levels of known substrates of PKA (P-Ser845 GluA1, P-Thr34 dopamine-and cAMP-regulated phosphoprotein of 32 kDa [DARPP-32], and P-Ser133 phosphodiesterase 4 [PDE4]) or a downstream substrate of PKA (P-Thr202/Tyr204 extracellular signal-regulated kinase 2 [ERK2]) with or without incubation with SKF81297, a Drd1-like agonist.

In the vehicle-treated group (Fig. 4h–k and Additional file 1: Fig. S7), the effect of genotype on phosphorylation levels of all four substrates for PKA signaling was examined under both basal and SKF81297-stimulated conditions, suggesting that Drd1a-mediated PKA signaling is activated in Cn mutants. Interestingly, in the rolipram-treated group (Fig. 4l–o), phosphorylation levels of P-Ser845 GluA1 and P-Thr202/Tyr204 ERK2 were not significantly different between Cn mutants and controls (Fig. 4l and m), whereas levels of P-Thr34 DARPP-32 and P-Ser133 PDE4 remained increased in Cn mutants (Fig. 4n and o). In Cn mutants, chronic rolipram treatment shifted P-Ser845 GluA1 and P-Thr202/Tyr204 ERK2 levels downward, and there was a tendency of decrease in P-Ser845 GluA1 levels (*P* = 0.090 for P-Ser845 GluA1 and *P* = 0.13 for P-Thr202/Tyr204 ERK2) (Additional file 1: Fig. S8). In contrast, in control mice, P-Ser845 GluA1 and P-Thr202/Tyr204 ERK2 levels were shifted upward following chronic rolipram treatment, and the effect of rolipram treatment on P-Thr202/Tyr204 ERK2 levels was significant (*P* = 0.0070) (Additional file 1: Fig. S8). These results indicate that chronic rolipram treatment modulates PKA activity downstream of Drd1a in a substrate-selective manner with the opposite effect between Cn mutant and control mice. When we examined the dose-dependency curves induced by SKF81297 application using data normalized to the level of each group untreated with SKF81297, a similar increase was found in SKF81297-induced phosphorylation between genotypes in ERK2, DARPP-32, and PDE4, but not in GluA1 (Additional file 1: Fig. S9), suggesting that Drd1 signaling is partially disturbed in Cn mutant mice.

## Discussion

In this study, we found immaturity-related signatures in the DG of adult Cn mutant mice, as assessed by the expression of typical molecular markers for neuronal maturation and genome-wide gene expression patterns. Considering that deletion of the *Cnb1* gene mediated by Cre under the Camk2a promoter was not observed until postnatal 5 weeks of age [24], it is likely that the iDG phenotype appears thereafter in the Cn mutants. During postnatal mouse development, calbindin expression levels in the DG almost reached a plateau at 4 weeks of age when it did not change further [81]. Therefore, it is likely that Cn deficiency starting after 5 weeks of age may reverse the maturity of GCs, designated as dematuration [47]. A number of factors, including genetic [3, 4, 7] and non-genetic factors [5, 47, 82], have been suggested to postnatally induce dematuration of GCs. The iDG phenotype in Cn mutants could be another example of dematuration of GCs. The presence of a number of doublecortin- or calretinin-positive immature GCs in the middle and outer parts of the GC layer apart from the subgranular zone (a well-known neurogenic region) is suggested to be attributed to the dematuration of mature GCs rather than to adult neurogenesis [16]. 5-bromo-2′-deoxyuridine (BrdU) labeling assay indicated that adult neurogenesis was slightly but significantly increased in the DG of Cn mutants (manuscript in preparation), but no obvious changes were found in the number of GCs. Therefore, enhanced recruitment of new neurons might not be a major contributing factor to the iDG phenotype. However, it is unclear how Cn deficiency caused the iDG phenotype in mice, including whether it is mediated by a cell-autonomous or non-cell-autonomous role of Cn.

We should note the limitations of the present study. First, while we showed an increase in Drd1a expression in the DG of Cn mutants by qPCR analysis, it has not been determined whether the increase appears in GC. Spatial analysis of Drd1a expression in combination with specific GC markers using in situ hybridization will help address this issue. Second, pathway enrichment analysis showed that biological processes related to the development of particular cells/tissues were predominantly overrepresented in the DEGs of Cn mutants, which is in accordance with the iDG hypothesis in these mice. However, limitations of this analysis include the inequality in annotation between genes in the database used. We queried GO and MsigDB databases via BaseSpace platform. Commonly used databases for pathway analysis, including these, are basically created by the curation of published experimental data. Hence, the analyses using the databases tend to bias toward the already annotated genes in nature, while prior unannotated genes are ignored [83, 84]. Such annotation inequality bias of genes must be taken into consideration to interpret our results of pathway enrichment analysis. Importantly, targeted comparisons revealed the high similarities in the gene expression pattern of Cn mutants with that of typically developing infant mice and other established mice with iDG, which confirmed the iDG phenotype in Cn mutants in terms of global gene expression analysis, in addition to pathway enrichment analysis.

We found that chronic rolipram treatment rescued the deficits in nest-building behavior along with the iDG phenotype in Cn mutants. Nest-building behavior is considered to be a hippocampus-dependent behavior [78–80], and its deficits have been observed in multiple mouse models of neuropsychiatric disorders, including ASD, schizophrenia, and AD [85]. In our previous study, we showed that the rescue of the iDG phenotype was accompanied by the rescue of nesting behavior deficits in Hivep2 KO mice, a mouse model of schizophrenia and ID [3, 86]. These results suggest that the iDG may be involved in impaired nest-building behavior. However, while we focused on the DG in this study, the effect of *Cnb1* knockout by Camk2a-Cre appeared in other areas of the brain, indicating that functional and morphological neuronal abnormalities have been found in the areas, such as synaptic plasticity deficits as assessed by long-term depression [24] and an impaired short-wave ripple-associated replay [87] in the CA1 region of the hippocampus, impaired synaptic transmission induced by high-frequency stimulation and decreased high-frequency oscillatory activity assessed by γ oscillations in the prefrontal cortex [88], and the prevention of spine shrinkage suggested by a reduction in small spines in the prefrontal and visual cortices [27, 28]. In addition, considering that rolipram highly binds to the neocortex, cerebellum, and hippocampus in rodents [89], rolipram may exert its effects in brain areas other than the DG in Cn mutants. The present study did not address the causal link between iDG and impaired nesting behavior. Therefore, we cannot exclude the possibility that impaired nesting behavior is associated with brain abnormalities other than iDG alone or in combination with iDG. It would be of interest to examine whether rolipram treatment rescues the above-mentioned neuronal abnormalities other than iDG.

Under basal conditions, Cn mutants showed increased phosphorylation levels of PKA substrates (PDE4, ERK2, GluA1, DARPP-32, and CREB). P-Ser845 GluA1 is a substrate for Cn [90]. It has also been reported that Cn dephosphorylates P-Thr34 DARPP-32 [91], which inhibits protein phosphatase-1 (PP1) activity [92]. PP1 dephosphorylates GluA1 [93] and CREB [94] directly and indirectly decreases the phosphorylation of ERK2 [95]. Consistent with these observations, we observed that phosphorylation levels of DARPP-32, GluA1, ERK2, and CREB were increased in Cn mutants. In addition to this pathway, increased PKA activity downstream of Drd1 may facilitate CREB phosphorylation in Cn mutants. Phosphorylation of PDE4 at Ser133 increases its activity [96], which is thought to be a negative feedback mechanism that maintains intracellular cAMP levels. Increased Drd1 signaling and PDE4 phosphorylation may balance the production and degradation of cAMP at high levels in Cn mutants. However, CREB, DARPP-32, and ERK are known to be dephosphorylated by protein phosphatase 2A (PP2A) [97–99], which can be activated by PKA through phosphorylation of the B56δ subunit [100]. Studies are needed to examine activation/expression of PP2A, as well as PP1, to gain further insight into the molecular mechanism underlying the increased basal phosphorylation of these PKA substrates in Cn mutant mice.

The increased Drd1/PKA signaling in Cn mutants relative to that in controls disappeared after chronic rolipram treatment in a PKA substrate-dependent manner. Furthermore, chronic treatment with rolipram ameliorated the increased phosphorylation of CREB, a known PKA substrate. As rolipram is known to increase intracellular cAMP levels and hence activate PKA activity, our data would appear to be paradoxical. These changes are not accounted for by changes in Drd1a expression levels, since rolipram did not significantly affect its expression levels in either Cn mutants or controls. Considering that PKA activates PP2A [100], which is capable of directly dephosphorylating CREB, chronic rolipram administration might facilitate this pathway, resulting in decreased CREB phosphorylation levels in Cn mutants. Interestingly, previous studies have suggested some compensatory mechanisms for constitutive increases and decreases in intracellular cAMP levels by enhancing PDE activity [101] and increasing dopamine concentrations [102]. Based on our data and those of others, we speculate that chronic, sustained upregulation in Drd1a/cAMP/PKA signaling in the Cn mutant DG is a compensatory mechanism underlying the iDG and nesting behavior deficits; thus, facilitating this pathway chronically with rolipram administration might exert beneficial effects, resulting in the rescue of the iDG phenotype and impaired nest-building behavior. However, the exact molecular mechanisms underlying the paradoxical changes in PKA activity in response to rolipram in Cn mutants remain unclear. Other molecules that regulate cAMP levels and PKA activity, such as protein Gs, adenyl cyclase, PKA regulatory subunit, protein phosphatase, and PDE family members other than PDE4 may be involved in chronic rolipram-related changes in the DG.

While currently available antipsychotic medications are mainly defined by their ability to ameliorate the positive symptoms of schizophrenia, such as delusions and hallucinations, few therapeutic methods have been established for the negative symptoms and cognitive dysfunction of the disease [103–105]. In this study, we found that chronic rolipram treatment rescued the decrease in nest-building activity in Cn mutants. The effect of the drug on the behavior was nominally significant but failed to reach study-wide significance; hence, this finding needs to be replicated in different cohorts. We previously found that rolipram (in combination with ibuprofen) rescued the decreased nest building and iDG phenotype in Hivep2 KO mice [3]. Therefore, PDE4 could be considered an important target for the treatment of the phenotypes. Indeed, a pilot study has reported that rolipram treatment exhibited some improvement of symptoms observed in patients with schizophrenia; however, its clinical use is limited due to side effects, such as nausea and vomiting [102], which may be due to the broad action of rolipram on PDE subtypes [106]. The development of PDE4 inhibitors without side effects may be a potential novel treatment for negative symptoms associated with neuronal immaturity in the DG in neuropsychiatric disorders, including schizophrenia [107]. Note that chronic rolipram treatment did not rescue other behavioral abnormalities examined in Cn mutants (e.g., hyper-locomotor activity, impaired PPI, and working memory deficits), suggesting that different molecular bases may underlie each behavioral phenotype.

In conclusion, this study adds to the literature indicating that the iDG phenotype can be induced by many genetic and non-genetic factors and is a possible endophenotype commonly found in neuropsychiatric disorders, including schizophrenia, ID, ASD, epilepsy, and AD [21]. We consider that the iDG in Cn mutants could be a phenotypic model linking Drd1a-mediated hyperdopaminergic dysregulation and neurodevelopmental abnormalities and may serve as a potential therapeutic target, especially for negative symptoms of neuropsychiatric disorders.

## Supplementary Information

**Additional file 1:** Supplementary Materials and Methods. **Figure S1.** No significant differences in the expression of mature/immature granule cell markers in the DG among three genotypes used for control mice. **Figure S2.** Decreased expression of GluA1 and GluA2 in the molecular layer of DG in Cn mutant mice. **Figure S3.** Original blots for Fig. 1i. **Figure S4.** Increased expression levels of S100B in the DG of Cn mutant mice. **Figure S5.** No significant effects of chronic rolipram treatment on GluA1 or *Drd1a* expression in the DG of Cn mutant mice. **Figure S6.** No significant effects of chronic rolipram treatment on open field test, PPI, or T-maze spontaneous alteration task in Cn mutant mice. **Figure S7.** Original blots for Fig. 4, S8 and S9. **Figure S8.** Effect of chronic rolipram treatment on phosphorylation levels of PKA substrates in the DG of control and Cn mutant mice. **Figure S9.** Dose-dependent changes in PKA substrate phosphorylation levels by application of SKF81297.

**Additional file 2: Table S1.** Antibodies used in this study. **Table S2.** Raw data of RT-PCR used for analyses. **Table S3.** Differentially expressed genes (DEGs) in the DG of Cn mutant mice. **Table S4.** Pathway enrichment analysis of DEGs. **Table S5.** Comparison of gene expression patterns in the DG of adult Cn mutant mice with those of infant mice at different time points after birth.

## Data Availability

The microarray data generated during the current study are available in the GEO database under the accession number GSE175896 (https://www.ncbi.nlm.nih.gov/geo/).

## Abbreviations

AD: Alzheimer’s disease
ASD: Autism spectrum disorder
Camk2a: Calcium/calmodulin dependent protein kinase II alpha
cAMP: Cyclic adenosine monophosphate
Cn: Calcineurin
CREB: CAMP-dependent response element binding protein
DARPP-32: Dopamine-and cAMP-regulated phosphoprotein of 32 kDa
DEGs: Differentially expressed genes
DG: Dentate gyrus
Drd1: Dopamine D1 receptor
Dsp: Desmoplakin
ERK2: Extracellular signal-regulated kinase 2
GC: Granule cell
GFAP: Glial fibrillary acidic protein
GluA1: Glutamate receptor 1
Hivep2: Human immunodeficiency virus type I enhancer binding protein 2
Iba1: Ionized calcium-binding adaptor molecule 1
ID: Intellectual disability
iDG: Immature dentate gyrus
PDE4: Phosphodiesterase 4
PKA: CAMP/protein kinase A
PSA-NCAM: Polysialylated neural cell adhesion molecule
Tdo2: Tryptophan 2,3-dioxygenase
