# Supplementary material for: Forebrain-specific conditional calcineurin deficiency induces dentate gyrus immaturity and hyper-dopaminergic signaling in mice
Source: Mol Brain. 2022 Nov 22;15:94. doi: 10.1186/s13041-022-00981-0 (PMC9682671; doi:10.1186/s13041-022-00981-0)
Supplement: Supplementary file 1 — Additional file 1: Supplementary Materials and Methods. Figure S1. No significant differences in the expression of mature/immature granule cell markers in the DG among three genotypes used for control mice. Figure S2. Decreased expression of GluA1 and GluA2 in the molecular layer of DG in Cn mutant mice. Figure S3. Original blots for Fig. 1i. Figure S4. Increased expression levels of S100B in the DG of Cn mutant mice. Figure S5. No significant effects of chronic rolipram treatment on GluA1 or Drd1a expression in the DG of Cn mutant mice. Figure S6. No significant effects of chronic rolipram treatment on open field test, PPI, or T-maze spontaneous alteration task in Cn mutant mice. Figure S7. Original blots for Fig. 4, S8 and S9. Figure S8. Effect of chronic rolipram treatment on phosphorylation levels of PKA substrates in the DG of control and Cn mutant mice. Figure S9. Dose-dependent changes in PKA substrate phosphorylation levels by application of SKF81297. [file 13041_2022_981_MOESM1_ESM.docx]

**Forebrain-specific conditional calcineurin deficiency induces dentate gyrus immaturity and hyper-dopaminergic signaling in mice**

Hideo Hagihara, PhD^1^; Hirotaka Shoji, PhD^1^; Mahomi Kuroiwa, PhD^2^; Isabella A Graef, MD, PhD^3^; Gerald R Crabtree, MD, PhD^3^; Akinori Nishi, MD, PhD^2^; Tsuyoshi Miyakawa, PhD^1*^

*^1^ Division of Systems Medical Science, Center for Medical Science, Fujita Health University, Toyoake, Aichi 470-1192, Japan*

*^2^ Department of Pharmacology, Kurume University School of Medicine, Kurume, Fukuoka 830-0011, Japan*

*^3^ Department of Pathology, Stanford University of Medicine, Stanford, California 94305, USA*

*^*^ Corresponding author*

**Additional file 1:**

Supplementary Materials and Methods

Figures S1–S9

Supplementary References

**Supplementary Materials and Methods**

**Immunohistochemistry**

Mice were deeply anesthetized with isoflurane and transcardially perfused with PBS followed by 4% PFA in PBS. The brains were dissected, immersed overnight in the same fixative, and cryoprotected by sequential incubation in 10, 20, and 30% sucrose in PBS for 2–3 days each at 4°C. After cryoprotection, brains were embedded in Tissue-Tek optimal cutting temperature compound (Miles, Elkhart, IN), frozen, and cut into 10-μm-thick coronal sections using a microtome (CM1850; Leica Microsystems, Wetzlar, Germany). The sections were pre-incubated for 1 hour at room temperature in 5% skim milk in PBST and then incubated overnight at 4°C in PBS containing the primary antibodies.

**Real-time quantitative PCR**

First-strand cDNA was prepared from 2 μg of DNase I-treated total RNA using SuperScript III reverse transcriptase (Invitrogen, Carlsbad, CA). The expression of related genes was quantified using the SYBR green reagent (2× SYBR Green PCR Master Mix; Qiagen, Valencia, CA) following the instructions of the manufacturer. Quantitative PCR was performed using DNA Engine Opticon 2 Real-Time PCR Detection System (Bio-Rad, Hercules, CA) with conditions as follows: 15 min at 95°C, then 45 cycles of 15 sec at 94°C, 30 sec at 60°C, 30 sec at 72°C and 1 min at 65°C. β-actin was amplified from all samples to normalize expression.

**Immunoblot analysis**

The DG was dissected [1], snap-frozen in liquid nitrogen, and stored at −80°C until use. The frozen tissues were homogenized in lysis buffer (FNN0021, Thermo Fisher Scientific, Waltham, MA). Twenty μg of protein were separated by gel electrophoresis using NuPAGE 4%–12% Bis-Tris gels (Thermo Fisher Scientific) and transferred to polyvinylidene difluoride membranes. The membranes were preincubated in 5% bovine serum albumin in Tris-buffered saline with 0.05% Tween® 20 (TBST) for 1 hour at room temperature and incubated overnight at 4°C in primary antibody, rabbit polyclonal anti-CREB antibody or rabbit polyclonal anti-phospho-CREB antibody, diluted in TBST. Immunoreactivity was visualized with a chemiluminescence detection regent (ImmunoStar LD, 292-69903; FUJIFILM Wako Pure Chemical Corporation, Osaka, Japan) and photographed with a luminescent image analyzer (Amersham ImageQuant 800; Cytiva, Tokyo, Japan).

**Behavioral tests**

The behavioral tests were performed as described previously [2–4].

*Open field test:* The apparatus was a transparent square cage (42 cm (width) × 42 cm (depth) × 30 cm (height)) equipped with infrared photobeam sensors (VersaMax; Accuscan Instruments, Columbus, OH, USA) with a white floor. The center of the floor was illuminated at 100 lux. Each mouse was placed in the corner of the cage. The total distance traveled (cm), vertical activity (rearing measured by counting the number of photobeam interruptions), time spent in the center area (20 cm × 20 cm), and stereotypic counts (defined by the number of breaks of the same beam) were recorded for 120 minutes using the VersaMax system.

*T-maze test:* The spontaneous alternation task was conducted using an automatic T‐maze apparatus (O’HARA & Co.) The apparatus was constructed of white PVC plastic runways with 25‐cm high walls. The maze was partitioned off into six areas by sliding doors that can be automatically opened downward. The stem of the T was composed of area S2 (13 × 24 cm), and the arms of T were composed of areas A1 and A2 (11.5 × 20.5 cm). Areas P1 and P2 were the connecting passageways from the respective arm (area A1 or A2) to the start compartment (area S1). Mice were placed in the S1 area and immediately subjected to a spontaneous alternation protocol for three sessions, with one session a day. One session consists of 10 trials with a 60‐minute cutoff time. Each trial had first and second runs. On the first run, the mouse was forced to choose one of the arms of the T (area A1 or A2). After the mouse stayed more than 10 seconds, the door that separated the arm (area A1 or A2) and the connecting passageway (area P1 or P2) would be opened, and the mouse could return to the starting compartment (area S1) via the connecting passageway. The mouse was then given a 3‐second delay in area S1, followed by a free choice between both T arms. The percentage of trials in which mice entered the arm opposite to their forced‐choice run during the free choice run was calculated. The location of the sample arm (left or right) varied pseudo‐randomly across trials using the Gellermann schedule so that mice received equal numbers of left and right presentations. Data acquisition, control of sliding doors, and data analysis were performed by ImageTM software (see “Image Analysis” below).

*Startle response/prepulse inhibition (PPI) test:* A startle reflex measurement system (O’HARA & Co.) was used to measure the acoustic startle response and PPI. A test session began by placing a mouse in a transparent plastic cylinder where it was left undisturbed for 10 minutes. White noise (40 ms) was used as the startle stimulus for all trial types. The startle response was recorded for 400 ms starting with the onset of the startle stimulus. The background noise level in each chamber was 70 dB. A test session consisted of six trial types (i.e., two types for startle stimulus‐only trials, and four types for PPI trials). The intensity of the startle stimulus was 110 or 120 dB. The prepulse sound was given 100 ms before the startle stimulus, and its intensity was 74 or 78 dB. Four combinations of prepulse and startle stimuli were used (74‐110, 78‐110, 74‐120, and 78‐120 dB). Six blocks of the six trial types were presented in a pseudo‐random order, such that each trial type was presented once within a block. The average inter‐trial interval was 15 seconds (range 10‐20 seconds).

*Nest building test:* To test the individual nest building behavior, mice were housed individually in cages containing paper chip bedding and one square of pressed cotton, “Nestlets” (Ancare, Bellmore, NY). No other nesting material (e.g., wood or wool) was present. The following morning, the manipulation of the Nestlet and the constitution of the built nest were assessed according to a five-point scale as described previously [5]: (1) Nestlet not noticeably touched (more than 90% intact); (2) Nestlet partially torn (50–90% remaining intact); (3) Nestlet mostly shredded but with no identifiable nest site (less than 50% of the Nestlet remains intact, but less than 90% is within a quarter of the cage floor area (i.e., the cotton is not gathered into a nest but rather spread around the cage), with the material may sometimes in a broadly defined nest area, but, critically, with 50–90% shredded); (4) an identifiable but flat nest (more than 90% of the Nestlet is torn, the material is gathered into a nest within a quarter of the cage floor area, but the nest is flat with walls higher than mouse body height (of a mouse curled up on its side) for less than 50% of its circumference; and (5) a (near) perfect nest (more than 90% of the Nestlet is torn, and the nest is a crater, with walls higher than mouse body height for more than 50% of its circumference).

*Image analysis:* Behavioral data were obtained automatically through applications based on the ImageJ program, and they were modified for each test by Tsuyoshi Miyakawa (available through O’HARA & Co.). The ImageJ plugins, and the precompiled plugins for open field test (Image OF) and T‐maze (Image TM) are freely available on the website of “Mouse Phenotype Database” (http://www.mousephenotype.org/softw are.html) [6].

**Figure S1. No significant differences in the expression of mature/immature granule cell markers in the DG among three genotypes used for control mice.** Bar graphs show means ± SEM. Each dot represents one mouse. ^**^*P* < 0.01, one-way ANOVA followed by multiple comparison test. (a) Calb1 immunoreactivity (arbitrary unit, AU). 1 vs. 2: *P* = 0.99; 1 vs. 3: *P* = 0.71; 1 vs. 4: *P* < 0.0001; 2 vs. 3: *P* = 0.42; 2 vs. 4: *P* < 0.0001; 3 vs. 4: *P* < 0.0001. (b) GluA1 immunoreactivity. 1 vs. 2: *P* > 0.99; 1 vs. 3: *P* = 0.58; 1 vs. 4: *P* < 0.0001; 2 vs. 3: *P* = 0.60; 2 vs. 4: *P* < 0.0001; 3 vs. 4: *P* < 0.0001. (c) Number of PSA-NCAM-positive cells per area. 1 vs. 2: *P* > 0.99; 1 vs. 3: *P* > 0.99; 1 vs. 4: *P* < 0.0001; 2 vs. 3: *P* = 1.00; 2 vs. 4: *P* < 0.0001; 3 vs. 4: *P* < 0.0001. (d) Number of calretinin-positive cells per area. 1 vs. 2: *P* = 0.69; 1 vs. 3: *P* = 0.70; 1 vs. 4: *P* < 0.0001; 2 vs. 3: *P* > 0.99; 2 vs. 4: *P* < 0.0001; 3 vs. 4: *P* < 0.0001.

**Figure S2. Decreased expression of GluA1 and GluA2 in the molecular layer of DG in Cn mutant mice.** Quantified bar graphs of expression of GluA1 (a; t = 7.96, df = 22, *P* < 0.0001) and GluA2 (b; t = 10.53, df = 4, *P* = 0.00050) in the molecular layer. Bar graphs show means ± SEM. Each dot represents one mouse. Relative intensity means the ratio of the immunoreactive intensity obtained from the mutants to that of the controls. ^**^*P* < 0.01, Students t-test. Con, control mice; Mut, Cn mutant mice.

**Figure S3. Original blots for Figure 1i.** The chemiluminescence images of phospho-CREB (left) and CREB (right) merged with the bright-field image of colorimetric marker. C, control mice; M, Cn mutant mice.

**Figure S4. Increased levels of S100B expression in the DG of Cn mutant mice.** Immunostaining images and quantified bar graphs of S100B (molecular layer; left graph: t = 3.43, df = 10, *P* = 0.0064; right graph: t = 0.58, df = 10, *P* = 0.57). Bar graphs show means ± SEM. Each dot represents one mouse. Scale bar: 100 μm. Relative intensity means the ratio of the immunoreactive intensity obtained from the mutants to that of the controls. ^**^*P* < 0.01, Students t-test. Con, control mice; g, granule cell layer; h, hilus; m, molecular layer; Mut, Cn mutant mice.

**Figure S5. No significant effects of chronic rolipram treatment on GluA1 or *Drd1a* expression in the DG of Cn mutant mice.** (a) GluA1 expression in the molecular layer of DG assessed using immunohistochemistry (genotype effect: F(1, 10) = 13.12, *P* = 0.0047; drug effect: F(1, 10) = 0.16, *P* = 0.69; interaction: F(1, 10) = 0.12, *P* = 0.73). (b) *Drd1a* mRNA expression levels in the DG assessed using quantitative RT-PCR (genotype effect: F(1, 29) = 25.91, *P* < 0.0001; drug effect: F(1, 29) = 1.01, *P* = 0.32; interaction: F(1, 29) = 0.88, *P* = 0.36). Each dot represents one mouse. ^**^*P* < 0.01, two-way ANOVA followed by multiple comparison test. Rol, roliplam; Veh, vehicle.

**Figure S6. No significant effects of chronic rolipram treatment on open field test, PPI, or T-maze spontaneous alteration task in Cn mutant mice.** (a–d) Total distance traveled (a; genotype effect: F(1, 58) = 59.29, *P* < 0.0001; drug effect: F(1, 58) = 0.57, *P* = 0.45; interaction: F(1, 58) = 0.0091, *P* = 0.92), vertical activity (b; genotype effect: F(1, 58) = 78.83, *P* < 0.0001; drug effect: F(1, 58) = 5.79, *P* = 0.019; interaction: F(1, 58) = 0.019, *P* = 0.89), time spent in center area (c; genotype effect: F(1, 58) = 3.07, *P* = 0.085; drug effect: F(1, 58) = 4.06, *P* = 0.049; interaction: F(1, 58) = 0.097, *P* = 0.33), and stereotypic behavior counts (d; genotype effect: F(1, 58) = 19.71, *P* < 0.0001, drug effect: F(1, 58) = 0.86, *P* = 0.019; interaction: F(1, 58) = 0.030, *P* = 0.86) in the open field test. (e, f) Startle amplitude (e; 110 dB, genotype effect: F(1, 55) = 1.59, *P* = 0.21; drug effect: F(1, 55) = 1.67, *P* = 0.20; interaction: F(1, 55) = 0.042, *P* = 0.84; 120 dB, genotype effect: F(1, 55) = 0.024, *P* = 0.88, drug effect: F(1, 55) = 0.23, *P* = 0.63; interaction: F(1, 55) = 0.0012, *P* = 0.97) and percentage of prepulse inhibition (f; 74-110 dB, genotype effect: F(1, 55) = 4.40, *P* = 0.041; drug effect: F(1, 55) = 0.060, *P* = 0.81; interaction: F(1, 55) = 2.58, *P* = 0.11; 78-110 dB, genotype effect: F(1, 55) = 15.22, *P* = 0.0003; drug effect: F(1, 55) = 2.77, *P* = 0.10; interaction: F(1, 55) = 0.017, *P* = 0.90; 74-120 dB, genotype effect: F(1, 55) = 8.23, *P* = 0.0058; drug effect: F(1, 55) = 1.83, *P* = 0.18; interaction: F(1, 55) = 0.022, *P* = 0.88; 78-120 dB, genotype effect: F(1, 55) = 5.92, *P* = 0.018; drug effect: F(1, 55) = 3.13, *P* = 0.083; interaction: F(1, 55) = 0.20, *P* = 0.66). (g) Percentage of correct responses in the T-maze spontaneous alteration task (session 1, genotype effect: (F1, 56) = 4.19, *P* = 0.045; drug effect: F(1, 56) = 0.46, *P* = 0.50; interaction: F(1, 56) = 1.88, *P* = 0.18; session 2, genotype effect: (F1, 56) = 1.95, *P* = 0.17; drug effect: F(1, 56) = 1.29, *P* = 0.26; interaction: F(1, 56) = 0.63, *P* = 0.43; session 3, genotype effect: (F1, 56) = 0.49, *P* = 0.49; drug effect: F(1, 56) = 1.44, *P* = 0.24; interaction: F(1, 56) = 0.59, *P* = 0.44). Data represent the mean ± SEM. ^*^*P* < 0.05, ^**^*P* < 0.01, genotype effects in two-way ANOVA (a–f). N = 17, 19, 10, and 14 mice, respectively. Rol, roliplam; Veh, vehicle.

**Figure S7. Original blots for Figures 4, S8 and S9.**

**Figure S8. Effect of chronic rolipram treatment on phosphorylation levels of PKA substrates in the DG of control and Cn mutant mice.** Expression levels of P-Ser845 GluA1 (a, e), P-Thr202/Tyr204 ERK2 (b, f), P-Thr34 DARPP-32 (c, g), and P-Ser133 PDE4B1 (d, h) in the DG slices from control mice (Con, a­–d) and Cn mutant mice (Mut, e­–h) with or without preincubation with SKF81297. The data were normalized to total protein levels and values obtained in untreated slices from vehicle-treated control mice. ^#^*P* < 0.1, ^*^*P* < 0.05, ^**^*P* < 0.01, two-way ANOVA. (a) Genotype effect: F(1, 42) = 2.72, *P* = 0.11; effect of SKF81297: F(2, 42) = 14.65, *P* < 0.0001; interaction: F(2, 42) = 0.53, *P* = 0.59. (b) Genotype effect: F(1, 42) = 8.04, *P* = 0.0070; effect of SKF81297: F(2, 42) = 25.43, *P* < 0.0001; interaction: F(2, 42) = 0.52, *P* = 0.60. (c) Genotype effect: F(1, 42) = 0.91, *P* = 0.35; effect of SKF81297: F(2, 42) = 12.31, *P* < 0.0001; interaction: F(2, 42) = 0.93, *P* = 0.40. (d) Genotype effect: F(1, 36) = 50.39, *P* < 0.0001; effect of SKF81297: F(2, 36) = 8.33, *P* = 0.0011; interaction: F(2, 36) = 4.55, *P* = 0.017. (e) Genotype effect: F(1, 42) = 3.01, *P* = 0.091; effect of SKF81297: F(2, 42) = 11.04, *P* = 0.0001; interaction: F(2, 42) = 0.070, *P* = 0.93. (f) Genotype effect: F(1, 42) = 1.41, *P* = 0.13, effect of SKF81297: F(2, 42) = 15.87, *P* < 0.0001; interaction: F(2, 42) = 0.038, *P* = 0.96. (g) Genotype effect: F(1, 42) = 0.56, *P* = 0.46; effect of SKF81297: F(2, 42) = 13.20, *P* < 0.0001; interaction: F(2, 42) = 0.078, *P* = 0.93. (h) Genotype effect: F(1, 36) = 0.0031, *P* = 0.96; effect of SKF81297: F(2, 36) = 8.38, *P* = 0.0010; interaction: F(2, 36) = 0.41, *P* = 0.67. Data are shown as means ± SEM. Rol, roliplam; Veh, vehicle.

**Figure S9. Dose-dependent changes in PKA substrate phosphorylation levels by application of SKF81297.** Expression levels of P-Ser845 GluA1 (a, e), P-Thr202/Tyr204 ERK2 (b, f), P-Thr34 DARPP-32 (c, g), and P-Ser133 PDE4B1 (d, h) in the DG slices from vehicle (Veh)-treated mice (a–d) or rolipram (Rol)-treated mice (e–h) with or without preincubation with SKF81297. The data were normalized to total protein and values obtained with SKF81297-untreated slices for each of four groups (Con + Veh, Mut + Veh, Con + Rol, and Mut + Rol). (a) Genotype effect: F(1, 42) = 7.14, *P* = 0.017; effect of SKF81297: F(2, 42) = 47.81, *P* < 0.0001; interaction: F(2, 42) = 2.31, *P* = 0.11. (b) Genotype effect: F(1, 42) = 0.51, *P* = 0.48; effect of SKF81297: F(2, 42) = 34.89, *P* < 0.0001; interaction: F(2, 42) = 0.19, *P* = 0.83. (c) Genotype effect: F(1, 42) = 0.86, *P* = 0.36; effect of SKF81297: F(2, 42) = 17.08, *P* < 0.0001; interaction: F(2, 42) = 0.52, *P* = 0.60. (d) Genotype effect: F(1, 36) = 1.87, *P* = 0.18; effect of SKF81297: F(2, 36) = 8.02, *P* = 0.0013; interaction: F(2, 36) = 3.97, *P* = 0.028. (e) Genotype effect: F(1, 42) = 0.66, *P* = 0.42; effect of SKF81297: F(2, 42) = 46.21, *P* < 0.0001; interaction: F(2, 42) = 0.16, *P* = 0.85. (f) Genotype effect: F(1, 42) = 0.34, *P* = 0.57, effect of SKF81297: F(2, 42) = 20.55, *P* < 0.0001; interaction: F(2, 42) = 0.084, *P* = 0.92. (g) Genotype effect: F(1, 42) = 0.17, *P* = 0.69; effect of SKF81297: F(2, 42) = 14.51, *P* < 0.0001; interaction: F(2, 42) = 0.042, *P* = 0.96. (h) Genotype effect: F(1, 36) = 6.87, *P* = 0.013; effect of SKF81297: F(2, 36) = 14.39, *P* < 0.0001; interaction: F(2, 36) = 1.74, *P* = 0.19. Two-way ANOVA. Data are shown as means ± SEM.

**Supplementary References**

1. Hagihara H, Toyama K, Yamasaki N, Miyakawa T. Dissection of hippocampal dentate gyrus from adult mouse. J Vis Exp. 2009:1543.

2. Takao K, Kobayashi K, Hagihara H, Ohira K, Shoji H, Hattori S, et al. Deficiency of schnurri-2, an MHC enhancer binding protein, induces mild chronic inflammation in the brain and confers molecular, neuronal, and behavioral phenotypes related to schizophrenia. Neuropsychopharmacology. 2013;38:1409–1425.

3. Miyakawa T, Leiter LM, Gerber DJ, Gainetdinov RR, Sotnikova TD, Zeng H, et al. Conditional calcineurin knockout mice exhibit multiple abnormal behaviors related to schizophrenia. Proc Natl Acad Sci. 2003;100:8987–8992.

4. Nakajima R, Takao K, Hattori S, Shoji H, Komiyama NH, Grant SGN, et al. Comprehensive behavioral analysis of heterozygous Syngap1 knockout mice. Neuropsychopharmacol Rep. 2019;39:223–237.

5. Deacon RM. Assessing nest building in mice. Nat Protoc. 2006;1:1117–1119.

6. Hattori S, Okumura Y, Takao K, Yamaguchi Y, Miyakawa T. Open source code for behavior analysis in rodents. Neuropsychopharmacol Rep. 2019;39:67–69.
